# Isotope pattern deconvolution for peptide mass spectrometry by non-negative least squares/least absolute deviation template matching

**DOI:** 10.1186/1471-2105-13-291

**Published:** 2012-11-08

**Authors:** Martin Slawski, Rene Hussong, Andreas Tholey, Thomas Jakoby, Barbara Gregorius, Andreas Hildebrandt, Matthias Hein

**Affiliations:** 1 Department of Computer Science, Saarland University, Saarbrücken, Germany; 2 Center for Bioinformatics, Saarland University, Saarbrücken, Germany; 3 Luxembourg Centre for Systems Biomedicine (LCSB), University of Luxembourg, Esch-sur-AlzetteLuxembourg; 4 Division for Systematic Proteome Research, Institute for Experimental Medicine, , Kiel, Germany; 5 Institut für Informatik, Johannes-Gutenberg -Universität, Mainz, Germany

## Abstract

**Background:**

The robust identification of isotope patterns originating from peptides being analyzed through mass spectrometry (MS) is often significantly hampered by noise artifacts and the interference of overlapping patterns arising e.g. from post-translational modifications. As the classification of the recorded data points into either ‘noise’ or ‘signal’ lies at the very root of essentially every proteomic application, the quality of the automated processing of mass spectra can significantly influence the way the data might be interpreted within a given biological context.

**Results:**

We propose non-negative least squares/non-negative least absolute deviation regression to fit a raw spectrum by templates imitating isotope patterns. In a carefully designed validation scheme, we show that the method exhibits excellent performance in pattern picking. It is demonstrated that the method is able to disentangle complicated overlaps of patterns.

**Conclusions:**

We find that regularization is not necessary to prevent overfitting and that thresholding is an effective and user-friendly way to perform feature selection. The proposed method avoids problems inherent in regularization-based approaches, comes with a set of well-interpretable parameters whose default configuration is shown to generalize well without the need for fine-tuning, and is applicable to spectra of different platforms. The R package IPPD implements the method and is available from the Bioconductor platform (http://bioconductor.fhcrc.org/help/bioc-views/devel/bioc/html/IPPD.html).

## Background

Mass spectrometry (MS), often in conjunction with high performance liquid chromatography (HPLC), is the de-facto standard analytical tool to derive important biological knowledge about the protein content of whole cells, organelles, or biomedical samples like tumour or blood plasma. Within a typical experimental setup, purified proteins of the sample under study are digested by an enzyme. Before entering the mass spectrometer, peptides are separated chromatographically according to their physico-chemical properties in order to avoid a massive overlapping of peptide signals within a single scan. Nevertheless, due to the sheer number of peptides present in a sample, interfering patterns still occur frequently, not least because of post-translational modifications such as the deamidation of asparagines or glutamine residues. In order to obtain an unambiguous assignment of the signals, and in particular their isotope patterns, which is a prerequisite for a proper identification and quantification, every data point in *m*/*z* dimension is classified either as ‘signal’ or as ‘noise’ during the so-called feature detection phase. As this processing lies at the very root of every proteomic application, the quality of feature detection can have dramatic impact on the finally derived results and conclusions. In view of the large amount of data even a single MS experiment can produce, automated analysis is indispensable. However, due to various artifacts arising from electric and chemical noise and baseline trends, the identification of isotope patterns is error-prone and time consuming. In addition, severe overlaps of peptide signals within the same mass spectrometric scan can hamper a straightforward analysis furthermore. In recent years, numerous procedures have been developed to process this data (cf., e.g., [1-8]). Within this paper, we propose a novel method that is demonstrated to perform especially well in challenging situations, characterized e.g. by strong local variations in noise and intensity levels or the presence of isotope patterns of different charges exhibiting overlap, which in many cases may be difficult to resolve even for a human expert by visual inspection. Existing software typically depends on a large set of parameters requiring careful fine-tuning, often being rather sensitive to changes in the measurement process like the change of the platform, which makes a proper parameter choice a laboursome task. In contrast, the proposed method has been designed to depend on a comparatively small set of well-interpretable parameters whose default configuration is shown to be robust, yielding mostly excellent, but at least competitive performance on spectra of different platforms. In a nutshell, our method uses non-negative least squares or non-negative least absolute deviation regression to fit a spectrum *s* by a large dictionary of templates mimicking isotope patterns; since true positions and charges of isotope patterns in the spectrum are unknown in advance, regions where the signal exceeds a local measure of noise are identified and then a vast set of templates is placed in those regions. In the spirit of sparse recovery, a small subset of the templates, which reasonably explains the observed signal, is selected by applying hard thresholding with a locally adaptive choice of the threshold to the regression coefficients obtained previously. Our method is related to a formerly proposed template-based approach (NITPICK, [3]). As opposed to the present work, NITPICK uses *ℓ*_1_-regularized non-negative least squares. Without non-negativity constraints, this procedure is known as the lasso [9]. Reference [10] contains the first application of the lasso to the problem studied in the present paper. Given a dramatic increase in occurrence of high-dimensional datasets in recent years and the resulting need for feature selection, the lasso, due to computationally and theoretically appealing properties, has meanwhile become so popular that it can be regarded as a standard tool of modern data analysis [11]. In this respect, NITPICK follows the usual paradigm suggesting that *ℓ*_1_-regularization is the method of choice. In the present paper, we argue for a deviation from that paradigm mainly in view of the following two aspects. First, a major benefit of our fitting+thresholding approach is that parameter choice is more user-friendly, since the threshold can be interpreted in terms of a signal-to-noise ratio. This is unlike the regularization parameter of the lasso, which can in general not be related directly to the signal. In the presence of heterogeneous noise and model misspecifications, the ‘right amount’ of regularization is notoriously difficult to choose. Second, there is a substantial body of work showing that non-negativity constraints alone may suffice to recover a sparse target. Non-negative least squares + thresholding is analyzed in [12], where it is shown that it can significantly outperform the usual *ℓ*_1_-approach with respect to sparse recovery. See Section “Sparse recovery with non-negativity constraints: non-negative least squares + thresholding vs. the non-negative lasso” for a detailed discussion.

## Methods

A spectrum is understood as a sequence of pairs ${\left\{(x_{i},y_{i})\right\}}_{i=1}^{n}$, where *x*_*i*_=*m*_*i*_/*z*_*i*_ is a mass (*m*_*i*_, measured in Dalton *Da*) to charge (*z*_*i*_), and *y*_*i*_ is the intensity, i.e. the abundance of a particular mass (modulo charge state), observed at *x*_*i*_, *i*=1,…,*n*, which are assumed to be ordered increasingly.

### Template model

The ${\left(y_{i}\right)}_{i=1}^{n}=y$ are modeled as a positive combination of templates designed on the basis of prior knowledge about peak shape and composition of isotope patterns. If our model were perfectly correct, we could write

(1) $$y=\Phi \beta^{*}=\overset{C}{\underset{c=1}{\sum }}\Phi_{c}\beta_{c}^{*},\;\;\Phi_{c}=[\varphi_{c,1}\ldots \varphi_{c,p_{c}}],\;\;c=1,\ldots ,C,$$

where **Φ** is a non-negative matrix of templates and ***β***^∗^ is a non-negative coefficient vector. Both **Φ** and ***β***^∗^ can be arranged according to charge states *c*=1,…,*C*. Each sub-matrix **Φ **_*c*_ can in turn be divided into columns $\varphi_{c,1},\ldots ,\varphi_{c,p_{c}}$, where the entries of each column vector store the evaluations of a template *φ*_*c*,*j*_, *j*=1,…,*p*_*c*_, at the *x*_*i*_, *i*=1,…,*n*. It is assumed that only a small fraction of the templates in **Φ** are needed to represent the signal, i.e. ***β***^∗^ is highly sparse. The templates are of the form

(2) $$\varphi_{c,j}=\underset{k\in }{\sum }a_{c,j,k}\;\psi_{c,j,k,{휃}_{c,j}},$$

where the *ψ*_*c*,*j*,*k*_ are functions representing a single peak within an isotope pattern, depending on a location *m*_*c*,*j*_ and a parameter vector ***θ***_*c*,*j*_. In general, peaks can be modeled by Gaussian, Lorentzian, and sech^2^ shapes, cf. [13]. Due to their similarity, we restrict ourselves to the Gaussian, but provide in addition the exponentially modified Gaussian (EMG, cf., e.g., [14]), a model for a possibly skewed peak as occuring frequently in MALDI-TOF recordings, where late ion formation in the gas phase leads to tailed peaks [15]. The EMG is parameterized by ${휃}_{c,j}={\left(\alpha_{c,j},\sigma_{c,j},\mu_{c,j}\right)}^{⊤}\in R^{+}\times R^{+}\times R$ (for *α*_*c*,*j*_*↓*0, one obtains a Gaussian)

(3) $$\begin{array}{ll} \psi_{c,j,k}(x)=\frac{1}{\alpha_{c,j}}\exp\left(\frac{\overset{2}{\underset{c,j}{\sigma }}}{2\overset{2}{\underset{c,j}{\alpha }}}+\frac{\mu_{c,j}-(x-m_{c,j,k})}{\alpha_{c,j}}\right)\\ \;\times \left(1-F\left(\frac{\sigma_{c,j}}{\alpha_{c,j}}+\frac{\mu_{c,j}-(x-m_{c,j,k})}{\sigma_{c,j}}\right)\right),\\ \;F(t)=\int_{-\infty }^{t}\frac{1}{\sqrt{2\Pi }}\exp\left(-\frac{u^{2}}{2}\right)\;\mathrm{du.}\; & \; \end{array}$$

**Figure 1:**
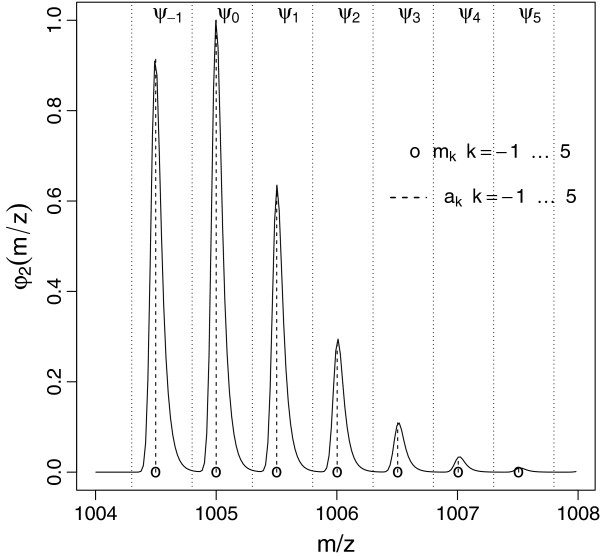
**Template model.** Illustration of the template construction (charge state *c*=2) for an EMG peak shape with a moderately strong right tailing.

In (2), the nonnegative weights *a*_*c*,*j*,*k*_ equal the height of the isotopic peak *k* within the pattern *j* of charge state *c*. These heights are computed according to the averagine model [16]. The *m*_*c*,*j*,*k*_ are calculated from *m*_*c*,*j*_ as $m_{c,j,k}=m_{c,j}+\kappa \frac{k}{c}$, where *κ* usually ranges between 1.002 and 1.008 Dalton, see e.g. [17]. Note that in Eq. (2) the location of the most intense peak (*a*_*c*,*j*,0_=max_*k*_*a*_*c*,*j*,*k*_) is taken as characteristic location of the template instead of using the finally reported monoisotopic position: we set *m*_*c*,*j*,0_=*m*_*c*,*j*_ so that the remaining *m*_*c*,*j*,*k*_, *k*≠0, are computed by shifting *m*_*c*,*j*_ in both directions along the *m*/*z* axis. With the normalization max_*x*_*φ*_*c*,*j*_(*x*)=1 for all *c,j*, the entries of ***β***^∗^ can be interpreted as intensities of the most intense peaks of the templates. The construction scheme is illustrated in Figure 1.

### Parameter estimation

The parameters *θ*_*c*,*j*_=(*α*_*c*,*j*_*σ*_*c*,*j*_*μ*_*c*,*j*_)^⊤^ of the peaks (3) are unknown in practice. Following a central paradigm of our framework, which is to relieve the user of performing laboursome fine-tuning of parameters, we have developed a systematic procedure automatically providing estimates of these parameters, which is considerably more efficient and flexible than a grid search. For instance, the parameters may additionally depend on the *m*/*z*-position. Our framework for parameter estimation extends a conceptually similar approach in [18] designed for a Gaussian peak shape.

**Figure 2:**
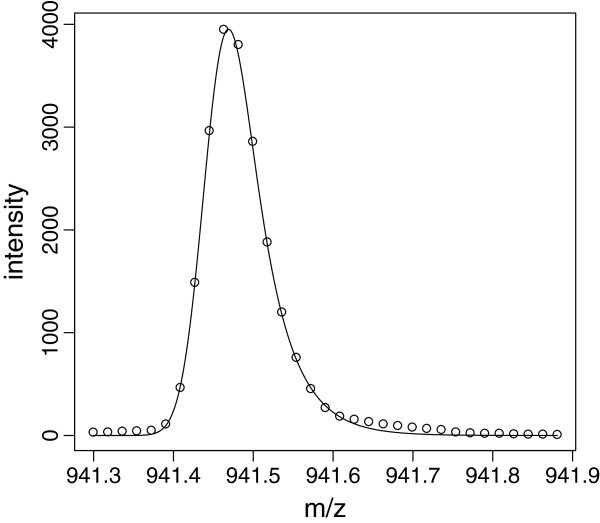
**Parameter estimation.** Illustration of peak parameter estimation. The figure displays a well-resolved peak in the region $R=\{i:\;941.3\;\mathrm{Th}\;\leq x_{i}\leq 941.9\;\mathrm{Th}\}$. In this example, the size of R equals 33, i.e. there are 33 pairs {(*x*_*i*_,*y*_*i*_)} that enter a nonlinear least squares problem of the form (4). Under the assumption of an EMG model, the resulting fit is indicated by a solid line.

In a first step, we apply a simple peak detection algorithm to the spectrum to identify disjoint regions $R_{r}\subset \{1,\ldots ,n\},\;\;r=1,\ldots ,R$, of well-resolved peaks. For each region, we fit the chosen peak shape to the data ${\left\{(x_{i},y_{i})\right\}}_{i\in R_{r}}$ using nonlinear least squares:

(4) $$\underset{휃}{\min}\underset{i\in R_{r}}{\sum }{\left(y_{i}-\psi_{휃}(x_{i})\right)}^{2},$$

yielding an estimate ${\hat{휃}}_{r}({\hat{x}}_{r})$, where ${\hat{x}}_{r}$ denotes an estimation for the mode of the peak in region $R_{r}$. This concept is sketched in Figure 2. The nonlinear least squares problem (4) is solved by using a general purpose nonlinear least squares routine available in most scientific computing environments, e.g. nls in R. Once the sequence of estimators $\left\{{\hat{휃}}_{r}({\hat{x}}_{r})\right\}$ has been obtained, they are subject to a suitable aggregation procedure. In the simplest case, one could simply take averages. For spectra where peak shape characteristics, in particular peak width, are known to vary systematically with *m*/*z* position, we use the pairs $\left\{({\hat{x}}_{r},{\hat{휃}}_{r}({\hat{x}}_{r}))\right\}$ as input into a linear regression procedure to infer the parameters of pre-specified trend functions. Formally, we model each component *θ*_*l*_ of ***θ*** as a linear combination of known functions *g*_*l*,*m*_ of *x*=*m*/*z* and an error component *ε*_*l*_, i.e.

(5) $${휃}_{l}(x)=\overset{M_{l}}{\underset{m=1}{\sum }}\nu_{l,m}g_{l,m}(x)+\varepsilon_{l}(x),$$

for which a linear trend i.e. *θ*_*l*_(*x*)=*ν*_*l*,1_ + *ν*_*l*,2_*x*, is one of the most common special cases. In [19], a set of instrument-specific models for the peak width is provided, all of which can be fitted by our approach.

We refrain from using least squares regression to determine the parameters in (5) due to its sensitivity to possible outliers, which arise from poorly resolved, wiggly or overlapping isotope patterns, which may affect the quality of the estimates ${\hat{휃}}_{r}$. Therefore, the linear model is fitted in a robust way by using least absolute deviation regression. Given the resulting estimates of the parameters {*ν*_*l*,*m*_}, *m*/*z*-specific estimates for the parameters in (3) are obtained by evaluating (5).

### Template fitting

The computation of the design matrix **Φ** requires a set of *m*/*z* positions at which templates are placed. In general, one has to choose positions from the interval [*x*_1_,*x*_*n*_]. We instead restrict ourselves to a suitable subset of the set ${\left\{x_{i}\right\}}_{i=1}^{n}$. The deviations from the positions of the true underlying isotope patterns is then at least in the order of the sampling rate, but this can be improved by means of a postprocessing step described in Section “Postprocessing and thresholding”. Using the whole set ${\left\{x_{i}\right\}}_{i=1}^{n}$ may be computationally infeasible if *n* is large and is in fact not necessary since isotope patterns occur very sparsely in the spectrum. Therefore, we apply a pre-selection step on the basis of what we term ‘local noise level’ (LNL). The LNL is defined as the median of the intensities *y*_*i*_ falling into a sliding window of fixed width around a specific position. For *x*∈[*x*_1_,*x*_*n*_], we define the local noise level based on sliding window width *h* as

(6) $$\begin{array}{ll} \text{LNL}(x)=\text{median}(\{y_{i}:\;i\in I_{x}\}),\;\\ {\;I}_{x}=\{i:\;x_{i}\in [x-h,x+h]\}.\; & \; \end{array}$$

Given the LNL, we place templates at position *x*_*i*_ (one for each charge state) if the corresponding *y*_*i*_ exceeds LNL(*x*_*i*_) by a factor factor.place. Section “Finding a set of default parameters” describes how we determined defaults for the two parameters *h* and factor.place. In fact, the LNL is a central quantity in our framework, because it does not only influence the placement, but also the selection of templates (see Section “Postprocessing and thresholding” below). Choosing *h* too small typically has the effect that the LNL is overestimated such that true peaks might be incorrectly classified as noise. Conversely, choosing *h* too large leads to an underestimation, thereby increasing the computational burden as well as the number of spurious patterns included in the final list. The advantages of working with the median are obvious: easy computation, robustness and equivariance with respect to monotone transformations. Similar notions of local noise can be found in the literature, see e.g. [8] where a truncated mean is used. Given the positions of the templates, we generate the matrix **Φ** according to Eqs. (1) and (2). In the fitting step, we compute a non-negative least squares (*q*=2) or alternatively non-negative least absolute deviation (*q*=1) fit by determining a minimizer $\hat{\beta }$ of the criterion

(7) $$\begin{array}{ll} \underset{\beta \geq 0}{\min}{∥y-\Phi \beta ∥}_{q}^{q},\;\;q=1\;\text{or}\;q=2, & \; \end{array}$$

The optimization problem (7) is a quadratic (*q*=2) or linear (*q*=1) program and is solved using interior point methods (e.g. [20]). The details are relegated to Appendix “Fitting with non-negativity constraints” section. As far as the choice of *q* is concerned, we point out that *q*=1 yields a robust fit that can deal better with deviations from model assumptions, i.e. deviations from the averagine model or from the peak model. However, in general, we are unable to provide any recommendation about how to choose *q*. Therefore, in our validation, both are evaluated.

#### Comparison with pepex

In prior work [21], subsequently referred to as ‘pepex’, non-negative least squares fitting is used as well. An important difference to our approach is that the matrix **Φ** is not constructed from the convolution of isotope distributions and peak shapes as described in Section “Template model”. Instead, peak detection is applied first to reduce the raw intensity data to peak clusters, a step that is usually referred to as centroiding. At the second stage, called de-isotoping, peak clusters are fitted by a design matrix containing isotope distributions themselves, not convolved versions. While the approach is computationally more attractive and avoids estimation of peak shape parameters (cf. Section “Parameter estimation”), the division into centroiding and de-isotoping may lead to poor performance for low resolution and noisy data, or in the presence of overlapping patterns. In these cases, peak detection is little reliable. In our template-based approach, there is no separation of centroiding and de-isotoping. It performs much better in the aforementioned cases, since it operates directly on the data and is hence less affected if single peaks of a pattern are difficult to detect. This reasoning is supported by our evaluation in Section “Results and discussion” as well as that in [3]. At the same time, our approach can in principle be applied to centroided spectra as well. In this case, the columns of the matrix **Φ** directly represent isotope distributions instead of isotopic patterns.

### Postprocessing and thresholding

**Figure 3:**
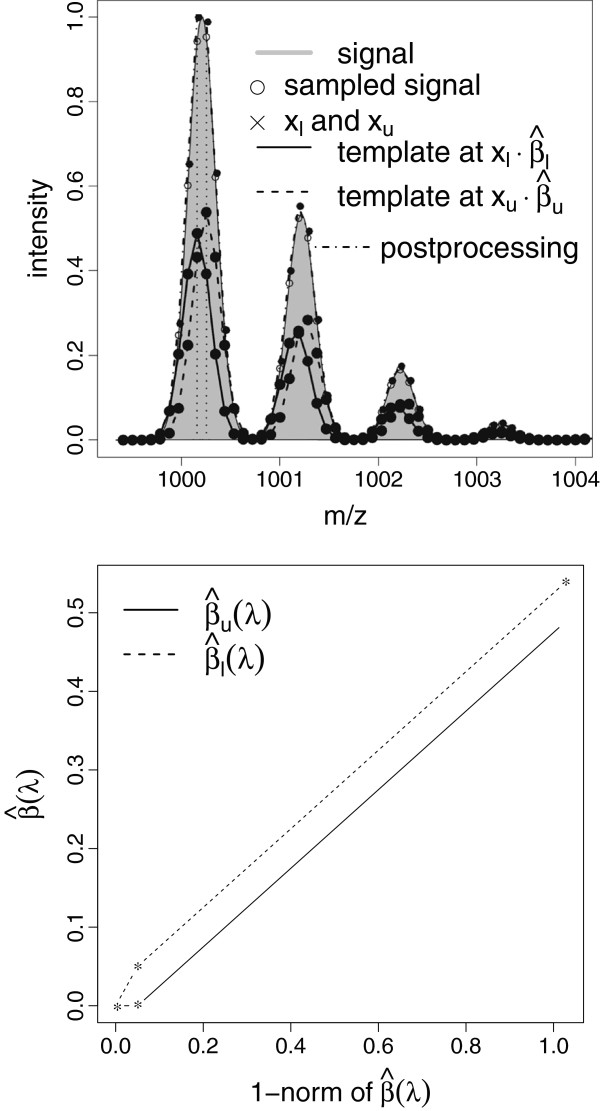
**Peak splitting.** Lower panel: Non-negative least squares fit of the sampled signal with and without postprocessing. Upper panel: Solution path of the non-negative lasso for the same data.

While indeed a considerable fraction of the entries of $\hat{\beta }$ are precisely equal to zero, treating all positions for which the corresponding entry differs from zero as locations of isotope patterns would yield a huge number of false positives, at least because of regions, in which noise fitting reduces the objective in (7). Therefore, the fitting step of the previous section is accompanied by a thresholding step, with the aim to separate signal from noise. However, fitting followed by thresholding alone does not lead to a proper output. The strategy could be successful if our template model were free of any kind of misspecification. Even when neglecting possible misfits of the averagine model, we still have to cope with two sources of systematic errors − a limited sampling rate and mismatches in the peak model. These are the main reasons for what we term ‘peak splitting’, referring to the phenomenon that several templates are used to fit precisely one pattern. Figure 3 illustrates the effect of sampling in a noiseless setting. In the top panel, the signal is sampled in such a way that the top of the peak is lost. When placing two templates at the two sampling points *x*_*l*_*x*_*u*_ of maximum signal, non-negative least squares fitting attributes weights ${\hat{\beta }}_{l},{\hat{\beta }}_{u}$ of roughly equal size to the templates. The postprocessing procedure outlined below yields a suitable correction. One might object that ‘peak splitting’ is a problem inherent in our entirely fitting-oriented approach (7) not incorporating any form of regularization. The bottom panel of Figure 3 shows the solution path of the non-negative lasso [22] given by $\{\hat{\beta }(\lambda ),\;\lambda \geq 0\},\;\;\hat{\beta }(\lambda )={\text{argmin}}_{\beta \geq 0}{∥y-\Phi \beta ∥}_{2}^{2}+\lambda 1^{⊤}\beta$. One obtains two nearly parallel trajectories, demonstrating that only a heavily biased fit, which would undesirably lead to the exclusion of additional smaller signals, could accomplish the selection of only one template.

To a large extent, ‘peak splitting’ can be corrected by means of the following merging procedure, which we regard as postprocessing of the fitting step (7) and which we apply prior to thresholding. Given an estimate $\hat{\beta }$, we define ${\hat{ℳ}}_{c}=\{m_{c,j}:\;{\hat{\beta }}_{c,j}>0\}\subset {\left\{x_{i}\right\}}_{i=1}^{n},\;\;c=1,\ldots ,C,$ as the set of all template locations where the corresponding coefficient exceeds 0.

1. Separately for each *c*, divide the sets ${\hat{ℳ}}_{c}$ into groups $G_{c,1},\ldots ,G_{c,G_{c}}$ of ‘adjacent’ positions. Positions are said to be adjacent if their distance on the *m*/*z* scale is below a certain tolerance ppm specified in parts-per-million, cf. Section “Finding a set of default parameters”. In the context of ‘peak splitting’, the templates at locations sharing the same group are assumed to fit precisely one true underlying peak.

2. With the notation of Eq. (2), for each *c*=1,…,*C*, and for *g*=1,…,*G*_*c*_, we solve the following optimization problem.

(8) $$\begin{array}{c} ({\tilde{m}}_{c,g},{\tilde{\beta }}_{c,g})=\underset{\beta_{c,g}}{\underset{m_{\text{c,g}}}{\text{argmin}}}\;\;\overset{\infty }{\underset{-\infty }{\int }}\;\;\;{\left(\underset{m_{c,j}\in G_{c,g}}{\sum }\;\;\;{\hat{\beta }}_{c,j}\psi_{m_{c,j}}\left(x\right)-\beta_{c,g}\psi_{m_{c,g}}\left(x\right)\right)}^{2}\;\;\mathrm{dx}, \end{array}$$

with the aim to find a location ${\tilde{m}}_{c,g}$ and a weight ${\tilde{\beta }}_{c,g}$ of the most intense peak $\psi_{{\tilde{m}}_{c,g}}$ within an isotope pattern *φ*_*c*,*g*_ approximating the fit of the most intense peaks $\left\{\psi_{m_{c,j}}:\;m_{c,j}\in G_{c,g}\right\}$ within the isotope patterns $\left\{\varphi_{c,j}:\;m_{c,j}\in G_{c,g}\right\}$ best in a least squares sense.

3. One ends up with sets ${\tilde{ℳ}}_{c}={\left\{{\tilde{m}}_{c,g}\right\}}_{g=1}^{G_{c}}$ and coefficients ${\left\{{\tilde{\beta }}_{c,g}\right\}}_{g=1}^{G_{c}},\;\;c=1,\ldots ,C$.

The additional benefit of solving (8) in step two as compared to the selection of the template with the largest coefficient within each group as proposed in [3] is that we are able to determine the location of the pattern even more accurately as predetermined by a limited sampling rate, since in (8) we optimize the location over a continuum. The optimization problem (8) can be solved fast and accurately by sampling the integrand on a fine grid of points and then solving a nonlinear least squares problem with optimization variables *m*_*c*,*g*_ and *β*_*c*,*g*_.

All candidate positions $\left({\tilde{m}}_{c,g},{\tilde{\beta }}_{c,g}\right)$ are assigned a signal-to-noise ratio

(9) $${\text{ratio}}_{c,g}={\text{GOF}}_{+}({\tilde{m}}_{c,g})\cdot \frac{{\tilde{\beta }}_{c,g}}{{\text{LNL}}_{+}({\tilde{m}}_{c,g})},$$

where ${\text{LNL}}_{+}({\tilde{m}}_{c,g})=\max\left\{\text{LNL}({\tilde{m}}_{c,g}),\frac{1}{4}\text{median}\;({\left\{\text{LNL}(x_{i})\right\}}_{i=1}^{n})\right\}$ is a truncated version of the local noise level, with a lower bound included to avoid that the denominator in (9) takes on tiny values in low-intensity regions. The factor ${\text{GOF}}_{+}({\tilde{m}}_{c,g})$ represents a goodness-of-fit adjustment, a correction which aims at downweighting spurious peaks in low-intensity noise regions. These are not hard to distinguish from signal regions, which, in view of the presence of peak patterns, tend to be considerably regular. In order to spot noise regions, we fit the spectrum by *single* peaks (3) placed at each datum *x*_*i*_, *i*=1,…,*n*, where the peak shape model, the associated peak shape parameters and the parameter *q* are chosen according to the choice made for template generation (Sections “Template model”) and template fitting (Section “Template fitting”), respectively. Denote the residuals of the resulting fit by ${\left\{r_{i}\right\}}_{i=1}^{n}$. A local measure of goodness-of-fit is defined by

(10) $${\text{GOF}}_{+}\left(x\right)=\min\left\{1-\frac{\underset{i\in I_{x}}{\sum }|r_{i}{|}^{q}}{\underset{i\in I_{x}}{\sum }|y_{i}{|}^{q}},0.5\right\},$$

The idea underlying this procedure is that in noise regions, the fit to the data will be poor, and consequently, the size of the residuals is expected to be large relative to the signal, hence leading to a low goodness-of-fit statistic. The truncation at 0.5 limits the influence of this correction. A final list is generated by checking whether the signal-to-noise ratios (9) exceed a ‘significance threshold’ *t* specified by the user. We do not give a general guideline for choosing *t*, because a reasonable choice is very specific to experimental conditions, e.g. the platform used and the composition of the spectrum. It is important to note that while *t* itself is constant, we take into account that the noise level is heterogeneous, since thresholding is based on the ratios (9), where the local noise level enters.

### Finding a set of default parameters

Apart from the signal-to-noise threshold *t*, we have introduced the parameters window, i.e. the width *h* of the sliding window required for the computation of the local noise level (6), the template placement parameter factor.place and the parts-per-million tolerance ppm within which peaks are considered to be merged by the postprocessing procedure. With the exception of the threshold *t*, we have fixed all parameters to a default setting which we expect to give reasonable (albeit potentially suboptimal) results on spectra different from the ones analyzed here, without the need of manual tuning. In order to find such a default setting, we performed a grid search using only one selected spectrum of those described in Section “Datasets” below. While our default setting, which can be found in the HTML manual of the R package IPPD, already performs well, we recommend to do such a calibration to optimize the performance of our method.

### Sparse recovery with non-negativity constraints: non-negative least squares + thresholding vs. the non-negative lasso

We believe that our preference for the first alternative is a major methodological contribution that has potential to impact related problems where non-negativity problems come into play. In the present section, we provide, at a high level, a series of arguments rooting in the statistics and signal processing literature that clarify our contribution and support our preference.

#### Linear models and usual paradigms in statistics

The fact that we favour non-negative least squares + thresholding may seem implausible since it questions or partially even contradicts paradigms about high-dimensional statistical inference. Consider the linear model

(11) $$y\approx \Phi \beta^{*},\;y\in R^{n},\;\Phi \in {â}^{n\times p},$$

which corresponds to model (1), where ‘≈’ is used instead of ‘=’ to account for stochastic noise or model misspecifications. Linear models of the form (10) have been and continue to be objects of central interest in statistical modelling.

• Classical work in statistics shows that under mild conditions if the number of sample *n* grows at a faster rate than the number of features *p*, the ordinary least squares estimator ${\hat{\beta }}^{\text{ols}}\to \beta^{*}$ (in probability) as *n*→*∞*.

• Since many contemporary datasets, like the MS datasets of the present paper, are characterized by a large *p*, which is of the same order as *n* or even larger, the first bullet has considerably lost relevance. Translated to MS datasets, it provides a statement about the case where the resolution tends to infinity. Therefore, modern statistical theory studies regimes in which *p* is allowed to grow at a faster rate than *n*, with a focus on results that hold for finite sample sizes. These results hinge on some sort of sparsity assumption on ***β***^∗^, the simplest being that ***β***^∗^ is zero except for some index set (support) of small cardinality. In this context, a multitude of results has been proved (see e.g. [23] for an overview) indicating that the lasso estimate ${\hat{\beta }}^{\text{lasso}}$ is a statistically optimal procedure in the sense that *if the regularization parameter is chosen in the right way*, the squared Euclidean distance $∥{\hat{\beta }}^{\text{lasso}}-\beta^{*}{∥}_{2}^{2}$ is nearly of the same order as that of an estimator one could construct if the non-zeroes of ***β***^∗^ were known.

The second bullet provides quite some justification for NITPICK, which is based on the lasso. However, as detailed below, the italicized part can be critical. On the other hand, there are several results that support our approach.

#### The power of non-negativity constraints

• It turns out that the non-negativity constraint ***β***≥***0*** imposed in non-negative least squares (NNLS) may lead to a drastically better performance than that of the *ordinary* least squares estimator in ‘large *p*’ situations provided **Φ** satisfies additional conditions. Roughly speaking, it is shown in [12] that if **Φ** has non-negative entries, which is fulfilled for the template matching problem of Section “Template model”, the NNLS estimator $\hat{\beta }$ does not overfit and is unique even in the singular case (*p*>*n*). These results indicate that NNLS may behave surprisingly well in a high-dimensional setup, without using *ℓ*_1_-regularization, which is often propagated in the literature as basically the only option ([24], Section 16.2.2).

• There are several recent papers [25-27] in the sparse recovery literature in which it is shown that a sparse, *non-negative* vector can be recovered from few linear measurements *n*≪*p*. In [12], these results are extended to a noisy setup. More specifically, it is shown that NNLS + thresholding can consistently recover ***β***^∗^ and its support. Very recently, using similar conditions as in [12], Meinshausen [28] has established several guarantees of NNLS in a high-dimensional setup.

One should bear in mind that the non-negativity constraints are essential for our approach. Thresholding the unconstrained ordinary least squares estimator ${\hat{\beta }}^{\text{ols}}$ in general leads to poor results in the ‘large *p*’ situation.

#### Shortcomings of *ℓ*_1_-regularization in theory

In [12], it is not only shown that NNLS + thresholding is a sound strategy to perform sparse recovery of a non-negative target, but also examples are given where the non-negative lasso is outperformed even if its regularization parameter is set to match theoretical results and regardless of whether subsequent thresholding as advocated in [29,30] is used or not. In particular, inferiority of the lasso arises in the presence of small, yet significantly non-zero entries in ***β***^∗^. These are specifically affected by the non-negligible bias of *ℓ*_1_-regularization [31]. It is important to note that the comparison in [12] does not contradict prior comparisons of the lasso (aka soft thresholding) and (hard) thresholding for *orthonormal designs* (** Φ **^⊤^** Φ **=***I***) in [32,33], where both approaches perform similarly well and non-negativity constraints are not particularly important. Orthonormal designs, which lead to greatly simplified estimation problem are not of interest in the context of the paper, since the template matrix **Φ** is far from being orthonormal.

#### Shortcomings of *ℓ*_1_-regularization in practice

The study in [12] is of more theoretical nature, since all constants of the problem, in particular the noise level, are known, so that the regularization parameter can be set in an optimal fashion. This can realistically not be accomplished in practice. Likewise, the information-theoretic criterion employed in [3] as well as the data-splitting approach of [34] rely on knowledge of the noise level, or a consistent estimate thereof, which is hard to obtain in the ‘large *p*’ situation [35]. In any case, the regularization parameter remains a quantity that is hard to grasp and hence hard to set for a practitioner, since it cannot be related directly to the signal. In contrast, the threshold *t* admits a straightforward interpretation.

Moreover, when using *ℓ*_1_-regularization, data fitting and model selection are coupled. While this is often regarded as advantage, since model selection is performed automatically, we think that it is preferable to have a clear separation between data fitting and model selection, which is a feature of our approach. Prior to thresholding, the output of our fitting approach gives rise to a ranking which we obtain without the necessity to specify any parameter. Selection is completely based on a single fit simply by letting the the threshold vary. On the contrary, if one wants to reduce the number of features selected by the lasso, one resets the regularization parameter and solves a new optimization problem. Note that it is in general not possible to compute the entire solution path of the lasso [22] for the MS datasets used for the present paper, where the dimension of **Φ** is in the ten thousands so that the active set algorithm of [22] is prohibitively slow. In this regard, model selection by thresholding is computationally more attractive.

## Results and discussion

For the assessment of the pattern picking performance, in total eight spectra generated by two different ionization methods, matrix assisted laser desorption/ionization (MALDI) and electrospray ionization (ESI), respectively, form the basis of the evaluation. While MALDI has been coupled to a time-of-flight (TOF) mass analyzer, ESI MS spectra have been recorded on both a linear ion trap (LTQ) and an Orbitrap mass analyzer. In addition, a series of spectra were prepared with the aim of investigating in detail the method’s performance in the presence of overlapping peptides.

### Datasets

For MALDI mass spectra (Additional file 1), time of flight mass analysis was performed; spectra were recorded on an ABI MALDI-TOF/TOF 4800 instrument in positive ion mode using *α*-cyano-4-hydroxy-cinnamic acid (CHCA) as matrix. Nanospray ESI spectra (Additional file 2) were measured in positive ion mode on a Thermo LTQ Orbitrap Velos MS; both high resolution measurements using the Orbitrap mass analyzer (referred to as ‘Orbitrap’) and, alternatively, low resolution linear ion trap (IT) measurements were performed with this setup. This experiment has been chosen in order to demonstrate the utility of our method at different concentration levels, that it is robust with respect to changes in the data-generating process and that the method is capable of handling singly charged ions, the main form generated by MALDI MS, as well as higher charged ions formed in ESI MS. Tryptic digests (performed in 40 mM ammonium bicarbonate) of model proteins were used as analytes: bovine myoglobin and chicken egg lysozyme (10 and 500 fmol each) for MALDI-TOF experiments, and lysozyme (250 and 1000 fmol) for ESI experiments. Disulfide bonds were reduced with dithiothreitol (DTT) prior to alkylation, free cysteine residues were alkylated by iodacetamide. No further sample pretreatment was performed prior to MS analysis. When referring to these spectra, we omit that tryptic digests are given: e.g., the term ‘MALDI-TOF myoglobin spectrum (500 fmol)’ means the respective tryptic digest.

To demonstrate explicitly the method’s ability to separate strongly overlapping patterns even in the case of badly resolved signals, 22 additional spectra have been generated in positive ion mode on a Bruker Daltonics HCT Ultra Ion Trap MS with an electrospray ion source. Three synthetic peptides (cf. Section “Unmixing of overlaps” for details) with sequences corresponding to tryptic peptides from bovine serum albumin (BSA) were used as analytes. In each measurement two out of three peptides were mixed in different ratios to get overlapping peptide signals, also with different charge states. Two different concentrations (500 fmol/*μ*l and 1000 fmol/*μ*l) were injected into the mass spectrometer via a Cole-Parmer syringe pump.

### Validation strategy

Validation of pattern picking is notoriously difficult, because a gold standard which is satisfactory from both statistical and biological points of view is missing. In this context, a major problem one has to account for is that spectra frequently contain patterns whose shape is not distinguishable from those of peptides, but which are in fact various artifacts resulting e.g. from impurities during sample preparation and measurement. These artifacts do not constitute biologically relevant information and are, in this sense, ‘false positives’. An important instance are signals derived from the matrix (or from matrix-clusters) frequently observed in MALDI MS. The pattern of these signals is similar to that of peptides; nevertheless, due to their molecular composition, which differs significantly from that of an average peptide, the exact masses can be used to exclude these signals from the data analysis. On the other hand, from a statistical perspective which judges a method according to how well it is able to detect specific patterns in a given dataset, a qualification as ‘true positive’ is justified. With the aim to unify these aspects, we have worked out a dual validation scheme. In order to reduce the number of artifacts, all automatically generated lists of candidates for peptide masses as well as the lists of a human expert (see below) are postprocessed by a peptide mass filter [36]: only peptides whose monoisotopic mass deviated less than 200 ppm from the closest peptide mass center^a^ are used for subsequent evaluation.

#### Comparison with manual annotation

The first part investigates how well a method is able to support a human expert who annotates the spectra manually. More specifically, the automatically generated lists are matched to the manual annotation such that an entry of the list (potential peptide mass) is declared ‘true positive’ whenever there is a corresponding mass in the manual annotation deviating by no more than *Δ* ppm. Otherwise, it is declared ‘false positive’. In order to adapt *Δ* ppm to the resolution of the different mass lines, we used the following strategy: assuming that most of the peptides will have a mass larger than 700 Da, we determined the spacing *Δ*_*m*/*z*_ between neighboring data points in *m*/*z* direction for each mass spectrum in the lower mass range. If we further assume that a simple manual annotation by visual inspection can result in a mass deviation from the ‘correct’ mass position of at most *Δ*_*m*/*z*_, we can derive the following tolerance values: *Δ*=100 ppm for ion trap recordings, *Δ*=50 ppm in the case of MALDI-TOF recordings^b^ and *Δ*=20 ppm for Orbitrap data.

As the performance of our as well as those of all competing methods depends on a threshold-like parameter governing, crudely speaking, the trade-off between precision and recall, we explore the performance for a range of reasonable parameter values, instead of fixing an (arbitrary) value, which we believe to be little meaningful. The results are then visualized as ROC curve, in which each point in the (Recall, Precision)-plane corresponds to a specific choice of the parameter. Formally, we introduce binary variables {*B*_*i*_(*t*)} for each mass *i* contained in the list of cardinality $\hat{L}(t)$ when setting the threshold equal to *t*, where *B*_*i*_(*t*) equals 1 if the mass is matched and 0 otherwise, and denote by *L* the number of masses of the manual annotation. The true positive rate (recall, *R*), and the positive predictive value (precision, *P*) associated with threshold *t* are then defined by $R(t)=\frac{\underset{i}{\sum }B_{i}(t)}{L}$, $P(t)=\frac{\underset{i}{\sum }B_{i}(t)}{\hat{L}(t)}$. An ROC curve results from a sequence of pairs {*R*(*t*),*P*(*t*)} for varying *t*.

#### Database query

The second part evaluates the lists in terms of a query to the Mascot search engine [37], version 2.2.04. In particular, we account for a major problem of a manual annotation, namely that peptides yielding weak MS signals might easily be overlooked, but might be detected by methods designed to extract those weak signals. Since we are especially interested in demonstrating the method’s ability to separate overlapping patterns, we adapted the standard search parameters of Mascot’s peptide mass fingerprint routine to allow two missed cleavage sites and to incorporate the following (variable) post-translational modifications: ‘Oxidation (M)’, ‘Carbamidomethyl (C)’, ‘Amidated (Protein C-term)’, ‘Deamidated (NQ)’. In particular, the latter two modifications will frequently trigger MS signals interleaving with the pattern of their unmodified counterpart: in the case of a deamidation the modified ion shows a mass of approx. 0.98 Da more compared to the amidated peptide. The same mass tolerances as for the manual annotation are used. As for the comparison with the manual annotation, we evaluate several lists corresponding to different choices of the threshold. Instead of an ROC curve, which turned out to be visually unpleasant, we display the statistics (score, coverage and fraction of hits) of two lists per method, namely of those achieving the best score and the best coverage, respectively. The complete set of results as well as further details of our evaluation like the manual annotation are contained in Additional file 3.

### Competing methods

We compare our method in its two variants depending on the choice of the fitting criterion (cf. Eq. (7)), labelled *l*_1_ (*q*=1) and *l*_2_ (*q*=2), respectively, with the following competing methods.

#### Lasso

The ‘lasso’ method in this paper serves as surrogate for NITPICK. Since the ‘lasso’ is embedded into our framework while implementing a methodology that closely resembles NITPICK, we use the ‘lasso’ for the sake of convenience, to avoid an involved parameter optimization for NITPICK. Our lasso implementation benefits from the improved merging procedure of Section “Postprocessing and thresholding”. To accomodate a heterogeneous noise level, NITPICK divides spectra into bins. This can be avoided by determining a minimizer $\hat{\beta }(\lambda ;W)$ of the weighted non-negative lasso problem

(12) $$\begin{array}{ll} \underset{\beta \geq 0}{\min}∥y-\Phi \beta {∥}_{2}^{2}+\lambda 1^{⊤}W\beta ,\;\;\lambda >0, & \; \end{array}$$

where ***W*** is a diagonal matrix with entries *w*_*c*,*j*_=LNL_ + _(*m*_*c*,*j*_), *j*=1,…,*p*_*c*_, *c*=1,…,*C*, whose purpose is to re-scale the amount of *ℓ*_1_-regularization according to the local noise level. The columns of the template matrix **Φ** in (8)). The parameter *λ* here plays the role of the threshold *t*, cf. Section “Validation strategy”.

#### Pepex

As discussed in Section “Template fitting”, pepex performs centroiding and de-isotoping separately. De-isotoping is based on non-negative least squares. Since pepex is limited to detect patterns of charge state one, its performance is only assessed for MALDI-TOF spectra. Accordingly, when comparing the ouptput of pepex with the manual annotation, the few patterns of charge state two are excluded. The parameters nm, pft, mincd, maxcd and nsam were set to optimize performance with respect to manual annotation. The ROC curves are based on peaklists resulting from ten different choices of the signal-to-noise parameter snr.

#### Isotope wavelet

As opposed to our method, this approach is not able to handle overlaps. On the other hand, it typically shows strong performance in noisy and low intensity regions or on datasets with extremely low concentrations [39,40]. While the isotope wavelet is not limited to charge one, it is run in charge one only mode for the MALDI-TOF spectra, to achieve more competitive performance. For the sake of fair of comparison, the result of the isotope wavelet on the MALDI-TOF spectra are evaluated in the same way as those of pepex.

#### Vendor

The parameter setting for the ABI MALDI-TOF/TOF MS software was as follows: Local Noise Width (*m*/*z*) 250, Min Peak Width at FWHM 2.9. The Cluster Area Optimization S/N threshold has been dynamically adapted to about three times the S/N threshold as suggested by the ABI documentation. Since the vendor software is limited to charge one, its outputs are evaluated in the same way as those of pepex. Given the disproportionally high effort needed to find an optimal parameter setting of the vendor software for ESI spectra, its performance is not assessed.

### Results

#### Manual annotation vs. database query

**Figure 4:**
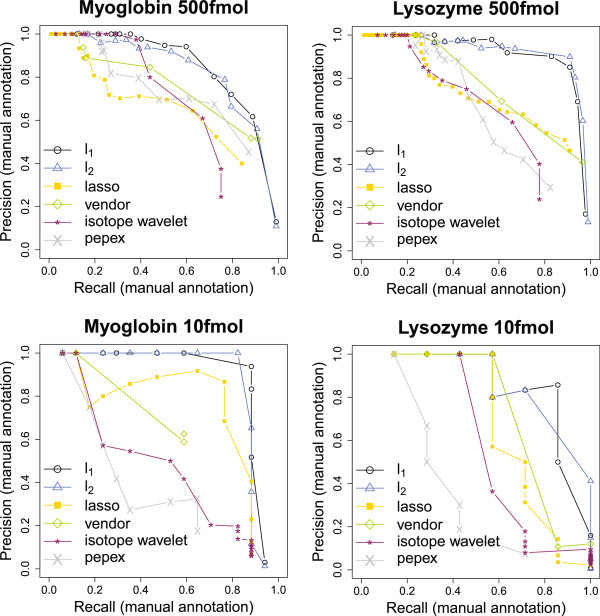
**Results for pattern picking, MALDI-TOF.** Pattern picking performance for the MALDI-TOF spectra as described in Section “Datasets”. The points in the (Recall,Precision)-plane correspond to different choices of a method-specific threshold(-like) parameter.

**Figure 5:**
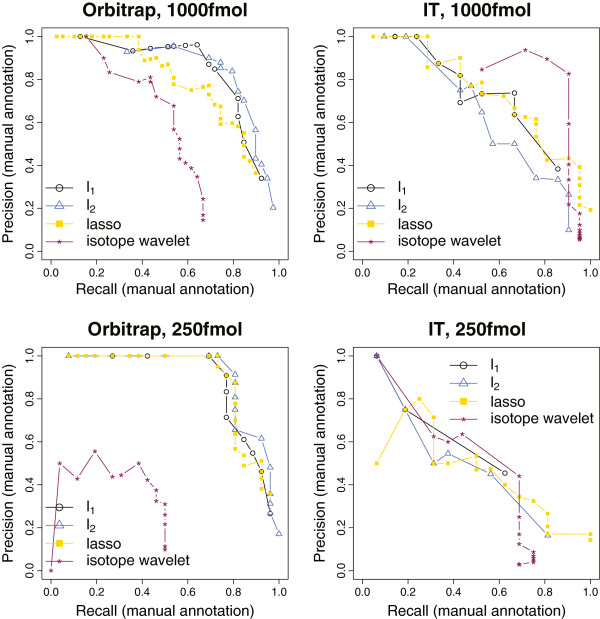
**Results for pattern picking, ESI.** Pattern picking performance for the ESI spectra as described in Section “Datasets”. The points in the (Recall,Precision)-plane correspond to different choices of a method-specific threshold(-like) parameter.

When inspecting Figures 4 and 5 on the one hand and Table 1 on the other hand, one notices that results of the evaluation based on the manual annotation are not in full accordance with the results of the database query. The difference is most striking for the MALDI-TOF spectra at 500 fmol, where our methods (*l*_1_ and *l*_2_) yield a significant improvement, which does not become apparent from the database query. This is because only a fraction of the manual annotation is actually confirmed by the database query. The part which is not matched likely consists of artifacts due to contamination or chemical noise as well as of specific modifications not captured by the database query. In light of this, our dual validation scheme indeed makes sense.

**Table 1:** Mascot results

**MALDI Myo 500 fmol**	**score**	**cvrg**	**hits**	**score**	**cvrg**	**hits**
*l*_1_	211.0	**0.85**	0.94	96.8	**0.96**	0.04
*l*_2_	211.0	**0.85**	0.94	49.6	**0.96**	0.04
lasso	207.0	**0.85**	**1.00**	142.0	0.91	**0.37**
pepex	**223.0**	**0.85**	**1.00**	142.0	0.90	0.17
vendor	**223.0**	**0.85**	0.94	**174.0**	0.90	0.29
wavelet	207.0	**0.85**	**1.00**	156.0	0.90	0.14
**MALDI Lys 500 fmol**	**score**	**cvrg**	**hits**	**score**	**cvrg**	**hits**
*l*_1_	167.0	**0.81**	0.57	133.0	**0.83**	**0.37**
*l*_2_	168.0	0.80	0.64	**144.0**	**0.83**	0.34
lasso	151.0	0.64	0.77	112.0	**0.83**	**0.37**
pepex	**172.0**	0.80	0.63	135.0	**0.83**	0.25
vendor	146.0	0.64	**0.75**	91.4	**0.83**	0.20
wavelet	127.0	0.58	**0.75**	113.0	0.81	0.20
**MALDI Myo 10 fmol**	**score**	**cvrg**	**hits**	**score**	**cvrg**	**hits**
*l*_1_	**211.0**	**0.85**	0.94	82.2	**0.95**	0.04
*l*_2_	207.0	0.74	**1.00**	109.0	0.90	0.14
lasso	195.0	0.77	0.87	**146.0**	0.85	0.46
pepex	97.8	0.80	0.22	97.8	0.80	0.22
vendor	123.0	0.62	0.62	123.0	0.62	**0.62**
wavelet	131.0	**0.85**	0.13	131.0	0.85	0.13
**MALDI Lys 10 fmol**	**score**	**cvrg**	**hits**	**score**	**cvrg**	**hits**
*l*_1_	**89.0**	0.35	**1.00**	**73.7**	0.54	**0.23**
*l*_2_	**89.0**	0.35	**1.00**	35.4	0.72	0.09
lasso	81.9	**0.46**	0.70	46.0	0.74	0.10
pepex	47.1	0.17	**1.00**	31.2	0.53	0.12
vendor	62.7	0.23	**1.00**	43.2	0.34	0.16
wavelet	55.4	0.23	0.45	43.8	**0.82**	0.10
**Orbi Lys 1000 fmol**	**score**	**cvrg**	**hits**	**score**	**cvrg**	**hits**
*l*_1_	149.0	0.70	0.78	138.0	0.80	**0.53**
*l*_2_	139.0	**0.80**	0.50	**139.0**	0.80	0.50
lasso	**159.0**	0.63	**0.87**	120.0	**0.81**	0.29
wavelet	105.0	0.69	0.44	95.1	0.80	0.23
**IT Lys 1000 fmol**	**score**	**cvrg**	**hits**	**score**	**cvrg**	**hits**
*l*_1_	78.7	0.63	0.28	70.9	0.74	0.17
*l*_2_	82.1	0.72	0.36	35.4	0.85	0.13
lasso	103.0	**0.84**	0.33	**76.8**	**0.99**	**0.21**
wavelet	**107.0**	0.79	**0.63**	69.8	**0.99**	0.11
**Orbi Lys 250 fmol**	**score**	**cvrg**	**hits**	**score**	**cvrg**	**hits**
*l*_1_	107.0	0.63	0.50	100.0	0.80	0.31
*l*_2_	103.0	0.63	0.52	66.9	**0.81**	0.14
lasso	**108.0**	0.63	**0.77**	**107.0**	0.80	**0.27**
wavelet	80.6	**0.70**	0.22	80.6	0.70	0.22
**IT Lys 250fmol**	**score**	**cvrg**	**hits**	**score**	**cvrg**	**hits**
*l*_1_	59.4	0.46	0.16	59.4	0.46	0.16
*l*_2_	37.0	0.59	0.14	37.0	0.59	0.14
lasso	**66.3**	**0.84**	0.20	**66.3**	**0.84**	**0.20**
wavelet	56.3	0.59	**0.36**	21.3	0.75	0.12

Corresponding Mascot results for the data shown in Figures 4 and 5. The left halves of the tables report the statistics when choosing the threshold(-like) parameter to optimize the score, the right halves when optimizing the coverage (cvrg, given as fraction). The column ‘hits’ contains the fraction of masses that could be matched to peptide masses in the database.

#### Comparison

Figure 4 and Table 1 reveal an excellent performance of our methods (*l*_1_ and *l*_2_) throughout all MALDI-TOF spectra under consideration. For the myoglobin spectra high sequence coverages are attained that clearly stand above those of competing methods. For the spectra at 10 fmol, only the performance of lasso is competetive with that of our methods in terms of the Mascot score; all other competitors, including the vendor software which has been tailored to process these spectra, are significantly weaker. In particular, the strikingly high proportion of ‘hits’ (≥94%) indicates that even at moderate concentration levels, our methods still distinguish well between signal and noise. This observation is strongly supported by the ROC curves in Figure 4, where the precision drops comparatively slowly with increasing recall. In this regard, our methodology clearly contrasts with approaches like the isotope wavelet that aim at achieving high protein sequence coverage. The latter often requires the selection of extremely lowly abundant peptide signals hidden in noise at the expense of reduced specificity.

For MALDI-TOF spectra at high concentration levels, pepex achieves the best scores and is competitive with respect to sequence coverage. However, the performance of pepex degrades dramatically at lower concentration levels, as it is unambiguously shown by both parts of the evaluation. In particular, the database scores are the worst among all methods compared. This provides some support for our reasoning at the end of Section “Template fitting”.

For the ESI spectra, our methods in total fall a bit short of the lasso (particularly for the ion trap spectra), but perform convincingly as well, thereby demonstrating that they can deal well with multiple charge states. This is an important finding, since the presence of multiple charges makes the sparse recovery problem as formulated in model (1) much more challenging, because the number of parameters to be estimated as well as the correlations across templates are increased. In spite of these difficulties, Figure 5 and Table 1 suggest that the performance of our pure fitting approach (7) does not appear to be affected. Using a more difficult set of spectra, the capability to process ESI data with impressive success is additionally shown in the next section.

#### Additional remarks

• In Figure 4, the area under the curve (AUC) of our methods attained for myoglobin is higher for lower concentration. At first glance, this may seem contradictory since an increase in concentration should lead to a simplified problem. However, a direct comparison of the AUCs is problematic, since the number of true positives (17 at 10 fmol, 106 at 500 fmol) is rather different. For instance, there are choices of the threshold that yield 18 true positives and not a single false positive for both of our methods at 500 fmol, yet the AUC is lower.

• The fact that some of the ROCs start in the lower left corner results from outputs containing only false positives.

### Unmixing of overlaps

#### Motivation

One of the main advantages of our method over more simplistic pattern picking methods is the ability to disentangle isotope patterns of overlapping peptide signals, whose presence may lead to a significantly more challening pattern picking problem as e.g. discussed in [41] in the slightly different context of intact protein mass spectra. Therefore, a potential application for our approach will be the analysis of a certain class of posttranslational modifications, the deamidation of amino acid residues containing a carboxamide side chain functionality. The deamidation of asparagine (Asn) or glutamine (Gln) residues, yielding aspartic acid (Asp) or glutamic acid (Glu) residues, respectively, is an important posttranslational modification, which can have immense effects on the structure of peptides [42] and is of great relevance in a number of pathophysiological events [43]. During the deamidation, the side chain carboxamide is hydrolysed, which is accompanied by a mass increase of 0.98 Da. Thus, in a spectrum of a mixture of the amidated and deamidated form, a direct overlap of both signals can be observed. It has to be noted that additionally to the amidated/deamidated forms, in case of Asn deamidation, a second product containing an iso-peptide bond is formed, too, which has the same molecular behaviour; these two forms can be identified solely by their differential MS/MS behavior.

#### Results

The peptides analyzed here in order to assess the performance of our approach were synthesized by means of Fmoc-solid phase peptide synthesis; sequences corresponding to tryptic peptides from bovine serum albumin (BSA) with the sequences listed in Table 2 were selected.

**Table 2:** Peptides mixed together

**sequence**	**sequence residueno. in BSA**	**monoisotopic mass(protonated) / charge**
GACLLPK	198-204	351.20437 / +2
CCTKPESER	460-468	351.48816 / +3
VLASSAR	212-218	352.20850 / +2

**Figure 6:**
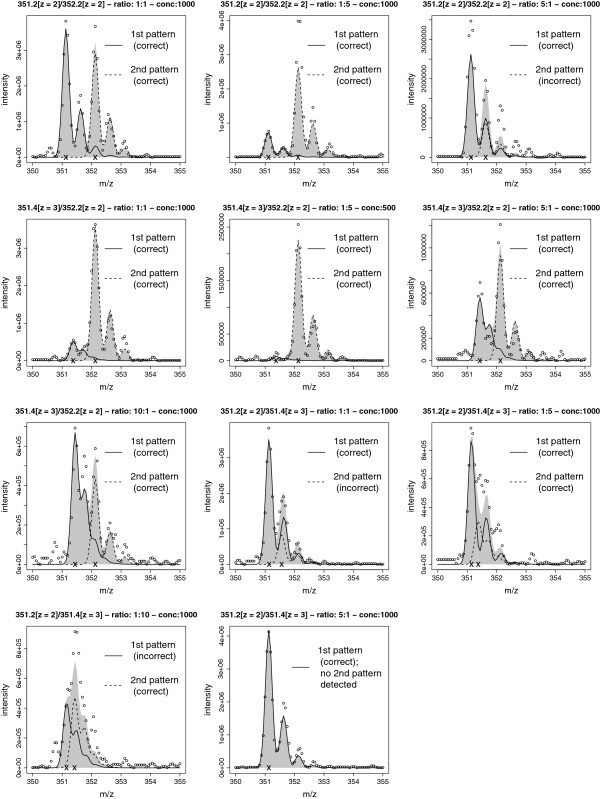
**Unmixing of overlap.** Graphical representation of selected overlap problems as tabulated in Table 3. The experimental setups are given in the title of the plots. The dots represent the signal, while solid and dashed lines represent the templates used by our method to match the signal, using the default parameter setting and the choice for the threshold as explained in the text. The grey area represents the overall fit when using the selected templates.

In each measurement two out of the three listed peptides were mixed together in different ratios (Additional file 4). Given such a spectrum, we study the question whether our method returns the true underlying composition. We classify the output of our method as correct interpretation of the spectrum if the templates corresponding to the true underlying peptides achieve signal-to-noise-ratios of at least one and these ratios are the two largest among all templates used for fitting. This procedure corresponds to a selection-optimal choice of the threshold based on the knowledge of the true composition of the spectrum. This simplification may be justified in view of the extreme difficulty of the problem as illustrated in Figure 6, in particular in view of lowly resolved spectra with an average *m*/*z*-spacing of 0.06 Da. For the remaining parameters, we compare a grid search (performed separately for each spectrum) and the default parameter set (Section “3 and Figure 6 indicate that already the default parameter setting is able to solve successfully a wide range of problem instances. As one would expect, Table 3 and Figure 6 suggest that the higher the concentration and the more balanced the amplitudes of the overlapping peptides, the more likely it is that the overlap can be resolved. On the other hand, the higher the degree of overlap of the peptides, which depends on both their charges and the distance of their positions, the more difficult the problem is. This becomes obvious when considering the overlap of the two peptides located at 351.2 and 351.4 Da, respectively.

**Table 3:** Unmixing of overlaps

**peptides**	**351.2(2)/352.2(2)**					**351.4(3)/352.2(2)**				**351.2(2)/351.4(3)**			
**proportion**	**1:1**		**1:5**	**5:1**	**1:1**	**1:5**	**5:1**	**10:1**	**1:1**	**1:5**	**5:1**	**1:10**	
	fmol											
	500	x	x	−	−	x	−	x	−	−	−	x
all	1000	x	x	x	x	x	x	x	x	x	−	−
	500	−	x	−	−	x	−	−	−	−	−	−
default	1000	x	x	−	x	−	x	x	−	x	−	−

Results of the analyses of the series of spectra containing two overlapping target peptides. The first column contains the *m*/*z* positions and the charges (in brackets) of the two peptides. The two middle columns indicate whether the corresponding problem is successfully solved (‘x’) or not (‘−’) when optimizing over a grid of parameter combinations (column ‘all’) and when using the default parameter set (column ‘default’).

## Conclusion

We have proposed a template matching approach for feature extraction in proteomic mass spectra. The main methodological innovation is a framework for sparse recovery in which sparsity is not promoted explicitly by a regularization term, as it is usually done and was done in previous work. We fully exploit the strength of non-negativity constraints, which permits us to circumvent the delicate choice of a ‘proper’ amount of regularization, an ever-lasting problem in statistics, and to work with thresholding instead. The latter is not only computationally attractive, because one does not have to repeatedly solve the same optimization problem for different choices of the regularization parameter, but also increases user-friendliness, since the threshold is directly related to the signal-to-noise ratio, the quantity domain experts are interested in. The replacement of a regularization parameter by a threshold is a cornerstone in our conceptual design guided by the principle to relieve the user from laboursome fine tuning of parameters. We believe that a small set of well-interpretable parameters with suitable defaults additionally improves robustness and reproducibility of results. In this context, we would like to emphasize again that apart from the threshold, the user does not have to specify any parameters before running our software.

In a comprehensive experimental study involving instruments of varying resolution and spectra of varying concentration levels, where we comparatively assess the performance of our approach on the basis of an elaborate dual validation scheme, it is demonstrated that the performance for pattern picking is excellent for MALDI-TOF spectra and outstands due to its specificity in selecting signal and only little noise. A major strength of the method is its ability to unmix overlapping peptide signals as shown for a series of ESI spectra. In total, we demonstrate that our approach is broadly applicable to a variety of spectra. While our approach is guided by a concrete application in proteomics, the framework is general enough to be of much of use for related deconvolution problems emerging in other fields − only the templates have to be adjusted according to the specific application.

While in this paper, we have focused on single spectra, the approach can be extended to process whole LC-MS runs, as it has already been implemented in our R package IPPD. More precisely, the sweep line scheme of [44] is used to agglomerate the results from single spectra. To apply our methods on a routine basis, an improved implementation, notably parallelization, is required, since e.g. processing a single spectrum of the Maxquant datasets [2] takes 10s on average on a Unix system equipped with an Intel(R) Core(TM)2 Duo CPU T9400 (2.53GHz) and 4 GB main memory. There is much room for an improvement, since our implementation is based on interpreted R code.

Concerning future directions of research, a question we have not yet answered in a satisfactory way is the choice of the fitting criterion. While both criteria (least squares and least absolute deviation) employed in this paper perform well, their implicit assumption of additive noise might be questionable [45]. It is worth investigating whether a multiplicative noise model could even yield better results. Second, one might ask whether the performance could be further improved when it is used jointly with the isotope wavelet, which is affected by overlaps, but has the potential to achieve higher protein sequence coverage.

## Endnotes

^a^Monoisotopic peptide mass centers are modelled by: 1.000485·*m*_*n*_ + 0.029, where *m*_*n*_ denotes the nominal mass.^b^For the MALDI-TOF lysozyme datasets an extended search tolerance of 100ppm was applied due to experimental miscalibration of the MS.

## Appendix

## Fitting with non-negativity constraints

In the following, we provide the details concerning optimization problem (7). In view of the special structure of **Φ **, (7) is computationally tractable even if *n* and the number of templates are in the ten thousands. We exploit the sparsity of the problem arising from templates which are highly localized, i.e. the domain on which they are numerically different from zero covers only a small part of the whole *m*/*z* range of the spectrum. As a consequence both **Φ** and the Gram matrix **Φ **^⊤^** Φ **, which is crucial in the computation, can conveniently be handled by using software for sparse matrices. For R, such software is available in the Matrix package [46].

### Non-negative least squares

Consider the quadratic program

(13) $$\begin{array}{ll} & \underset{\beta }{\min}\frac{1}{2}∥y-\Phi \beta {∥}_{2}^{2}\; \end{array}$$

(14) $$\begin{array}{ll} & \text{subject to}\;\beta \geq 0.\; \end{array}$$

In order to solve (12), we use the so-called log-barrier method which amounts to solving a sequence of an unconstrained nonlinear convex problems in which the constraints *I*(*β*_*j*_≥0), *j*=1,…,*p*, are taken into account by incorporating log-barrier terms −log(*β*_*j*_)/*γ* in the objective. As *γ*→*∞*, the log-barrier acts like a function which equals + *∞* if *β*_*j*_<0 and zero otherwise. Beginning with a moderately sized starting value for *γ*, we solve the convex problem

(15) $$\underset{\beta }{\min}\frac{1}{2}∥y-\Phi \beta {∥}_{2}^{2}-\frac{1}{\gamma }\overset{p}{\underset{j=1}{\sum }}\log(\beta_{j})$$

using Newton’s method. The gradient and Hessian with respect to ***β***, respectively, are given by

(16) $$\begin{array}{lll} \nabla_{\beta } & =-\Phi^{⊤}(y-\Phi \beta )-\frac{1}{\gamma }{\left[1/\beta_{1}\;\ldots \;1/\beta_{p}\right]}^{⊤}.\; & \;\\ \nabla_{\beta }^{2} & =\Phi^{⊤}\Phi +\frac{1}{\gamma }\text{diag}(1/\beta_{1}^{2},\ldots ,1/\beta_{p}^{2}).\; & \; \end{array}$$

The Newton descent direction ***d***_***β***_ is obtained from the linear system

(17) $$\nabla_{\beta }^{2}d_{\beta }=-\nabla_{\beta }.$$

Solution of linear systems of this structure constitutes the main computational effort to be made. Fast solutions are obtained by using CHOLMOD[47], which offers an efficient implementation for computing the Cholesky factorization of sparse symmetric, positive definite matrices. Since the diagonal of $\nabla_{\beta }^{2}$ changes from one Newton iteration to the next, one Cholesky factorization has to be performed per Newton step. Once we have solved (14) for a specific *γ*, we solve a new problem of the type (14) for *γ*·*M*, *M*>1. This is repeated until *γ* exceeds a predefined maximum value. For a thorough account on the log-barrier method, we refer to [20].

### Complexity analysis of non-negative least squares

We here provide the order of magnitude of floating points operations (flops) required per update (i.e. per Newton step) for the specific non-negative least squares problems considered for this paper. In our implementation, we exploit that the templates contained in the matrix **Φ** are highly localized. As a result, after a suitable column permutation, the matrix **Φ **^⊤^** Φ** is roughly a band matrix with bandwidth *k* no larger than only few hundreds. The dominant operation is solving the linear system $\nabla_{\beta }^{2}d_{\beta }=-\nabla_{\beta }$ with the help of the Cholesky factorization, which can be done in *O*(*pk*^2^) flops (e.g. [20], p.670). Our algorithm terminates after usually no more than one hundred Newton steps.

### Non-negative least absolute deviation

Consider the optimization problem

(18) $$\begin{array}{ll} & \underset{\beta }{\min}∥y-\Phi \beta {∥}_{1}\; \end{array}$$

(19) $$\begin{array}{ll} & \text{subject to}\;\beta \geq 0.\; \end{array}$$

Problem (15) can be recast as the following linear program.

(20) $$\begin{array}{lll} & \underset{r}{\min}r^{⊤}1\;\\ & \text{subject to}\; & \;\\ & \Phi \beta -y+r\geq 0,\; & \;\\ & y-\Phi \beta +r\geq 0,\; & \;\\ & r\geq 0,\; & \;\\ & \beta \geq 0.\; & \;\\ & \; \end{array}$$

For its solution, we use the log-barrier method sketched in the previous paragraph. After incorporating log-barrier terms for all constraints, the objectives of the unconstrained convex problems are of the form

(21) $$\begin{array}{c} r^{⊤}1-\frac{1}{\gamma }\;\left(\overset{n}{\underset{i=1}{\sum }}\left(\text{log},\left(\xi_{i}^{+}\right),+,\text{log},\left(\xi_{i}^{-}\right),+,\text{log},\left(r_{i}\right)\right)+\overset{p}{\underset{j=1}{\sum }}\log\left(\beta_{j}\right)\right), \end{array}$$

where we have used the notational shortcuts

(22) $$\begin{array}{lll} & \xi_{i}^{+}={\left(\Phi \beta \right)}_{i}-y_{i}+r_{i},\; & \;\\ & \xi_{i}^{-}=y_{i}-{\left(\Phi \beta \right)}_{i}+r_{i},\;\;i=1,\ldots ,\mathrm{n.}\; & \; \end{array}$$

The gradients w.r.t. ***r*** and ***β***, respectively, are given by

(23) $$\begin{array}{l} \nabla_{r}=1-\frac{1}{\gamma }{\left[\frac{1}{\left(\xi_{1}^{+}+\xi_{1}^{-}+r_{1}\right)}\;\ldots \;\frac{1}{\left(\xi_{n}^{+}+\xi_{n}^{-}+r_{n}\right)}\right]}^{⊤},\\ \nabla_{\beta }=-\frac{1}{\gamma }(\Phi^{⊤}({\left[\Xi^{+}\right]}^{-1}-{\left[\Xi^{-}\right]}^{-1})1+{\left[1/\beta_{1}\;\;\ldots \;\;1/\beta_{p}\right]}^{⊤}),\;\;\\ \Xi^{\pm }=\text{diag}(\xi_{1}^{\pm },\ldots ,\xi_{n}^{\pm }). \end{array}$$

Introducing ***R***=diag(*r*_1_,…,*r*_*n*_) and ***B***=diag(*β*_1_,…,*β*_*p*_), the Hessian is given by the block matrix

(24) $$\left[\begin{array}{ll} \nabla_{r}^{2} & \nabla_{r\;\beta }\\ \nabla_{r\;\beta }^{⊤} & \nabla_{\beta }^{2} \end{array}\right]=\left[\begin{array}{ll} \frac{1}{\gamma }({\left[\Xi^{+}\right]}^{-2}+{\left[\Xi^{-}\right]}^{-2}+R^{-2}) & \frac{1}{\gamma }({\left[\Xi^{+}\right]}^{-2}\Phi -{\left[\Xi^{-}\right]}^{-2}\Phi )\\ \frac{1}{\gamma }(\Phi^{⊤}{\left[\Xi^{+}\right]}^{-2}-\Phi^{⊤}{\left[\Xi^{-}\right]}^{-2}) & \frac{1}{\gamma }(\Phi^{⊤}({\left[\Xi^{+}\right]}^{-2}+{\left[\Xi^{-}\right]}^{-2})\Phi )+B^{-2}) \end{array}\right].$$

The linear system for the Newton descent directions reads

(25) $$\left[\begin{array}{cc} \nabla_{r}^{2} & \nabla_{r\;\beta }\\ \nabla_{r\;\beta }^{⊤} & \nabla_{\beta }^{2} \end{array}\right]\;\left[\begin{array}{c} d_{r}\\ d_{\beta } \end{array}\right]=-\left[\begin{array}{l} \nabla_{r}\\ \nabla_{\beta } \end{array}\right].$$

Note that $\nabla_{r}^{2}$ is diagonal, so it is a cheap operation to resolve for ***d***_***r***_ once ***d***_***β***_ is known:

(26) $$d_{r}=-{\left(\nabla_{r}^{2}\right)}^{-1}(\nabla_{r\;\beta }\;d_{\beta }+\nabla_{r}).$$

Plugging this into the second block of the linear system, one obtains

(27) $$-\nabla_{r\;\beta }^{⊤}{\left(\nabla_{r}^{2}\right)}^{-1}(\nabla_{r\;\beta }\;d_{\beta }+\nabla_{r})+\nabla_{\beta }^{2}d_{\beta }=-\nabla_{\beta }$$

which is equivalent to

(28) $$(\nabla_{\beta }^{2}-\nabla_{r\;\beta }^{⊤}{\left(\nabla_{r}^{2}\right)}^{-1}\nabla_{r\;\beta })d_{\beta }=-\nabla_{\beta }+\nabla_{r\;\beta }^{⊤}{\left(\nabla_{r}^{2}\right)}^{-1}\nabla_{r}.$$

In order to solve the linear system, we proceed as for non-negative least squares. The computational cost of this operation is roughly the same, since the sparse structure of **Φ **^⊤^** Φ** can still be exploited. For non-negative least squares, re-computation of the Hessian $\nabla_{\beta }^{2}$ only involves a diagonal update, an operation of negligible computational cost. However, for non-negative least absolute deviation, computation $\nabla_{\beta }^{2}$ involves the matrix multiplication (** Φ **^⊤^([***Ξ***^ + ^]^−2^ + [***Ξ***^−^]^−2^)** Φ **), i.e. essentially a repeated computation of a scaled Gram matrix. In spite of the special structure of **Φ **^⊤^** Φ **, the computational cost is of the same order as the solution of the linear system even when using a self-written routine for matrix multiplication tailored to the specific structure.

## Competing interests

The authors declare that they have no competing interests.

## Authors’ contributions

MS and MH devised the methodology as presented in Section “Methods”. MS implemented the Bioconductor package, with contributions by RH and MH. The comparative data analysis was performed by RH, MS, MH and AH; RH and AH performed the MASCOT queries. AT developed the experimental design and provided an interpretation of the MS data. TJ and BG conducted the MS experiments and produced the results of the vendor software. All authors read and approved the final manuscript.

## Supplementary Material

Additional file 1 MALDI-TOF spectra. Click here for file

Additional file 2 ESI spectra. Click here for file

Additional file 3 Evaluation and results. Click here for file

Additional file 4 Overlapping peptide signals. Click here for file
