# Supplementary material for: Isotope pattern deconvolution for peptide mass spectrometry by non-negative least squares/least absolute deviation template matching
Source: BMC Bioinformatics. 2012 Nov 8;13:291. doi: 10.1186/1471-2105-13-291 (PMC3608065; doi:10.1186/1471-2105-13-291)
Supplement: Additional file 3 — Evaluation and results. [file 1471-2105-13-291-S3.zip › eval/manual_annotation/excludedregions/screenshots/esi/excludedreg_FT1000_3.pdf]

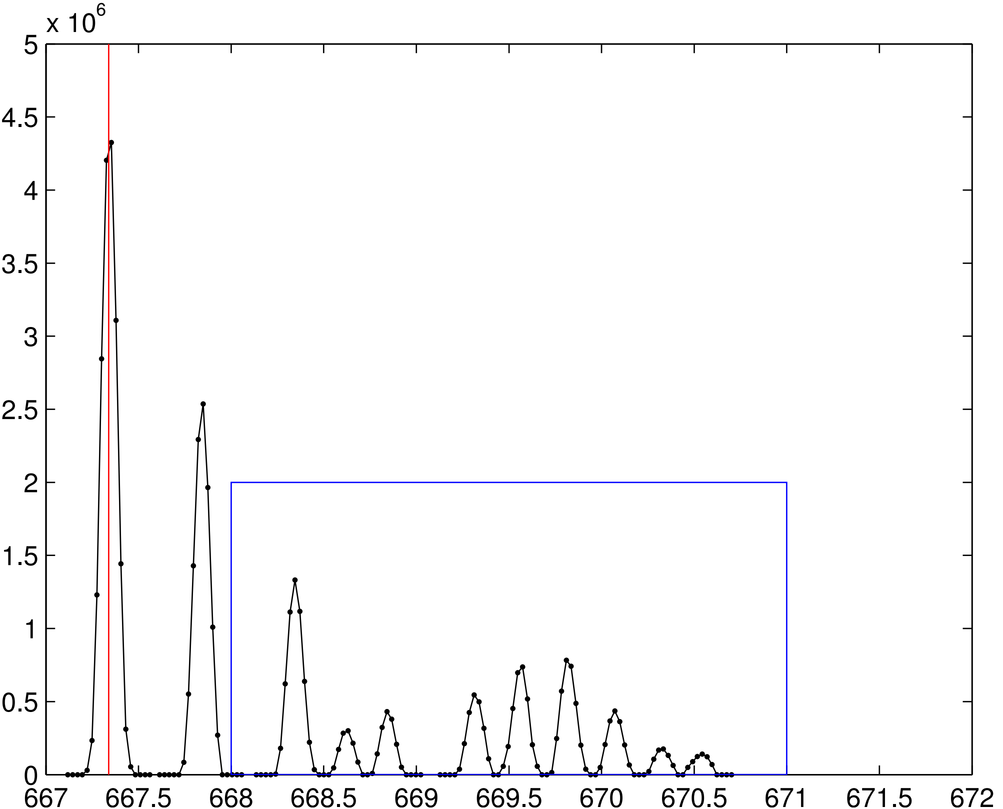

x 10^6
5
4.5
4
3.5
3
2.5
2
1.5
1
0.5
0
667
667.5
668
668.5
669
669.5
670
670.5
671
671.5
672
