# Supplementary material for: Isotope pattern deconvolution for peptide mass spectrometry by non-negative least squares/least absolute deviation template matching
Source: BMC Bioinformatics. 2012 Nov 8;13:291. doi: 10.1186/1471-2105-13-291 (PMC3608065; doi:10.1186/1471-2105-13-291)
Supplement: Additional file 3 — Evaluation and results. [file 1471-2105-13-291-S3.zip › eval/manual_annotation/excludedregions/screenshots/maldi/excludedreg_lys500_4.pdf]

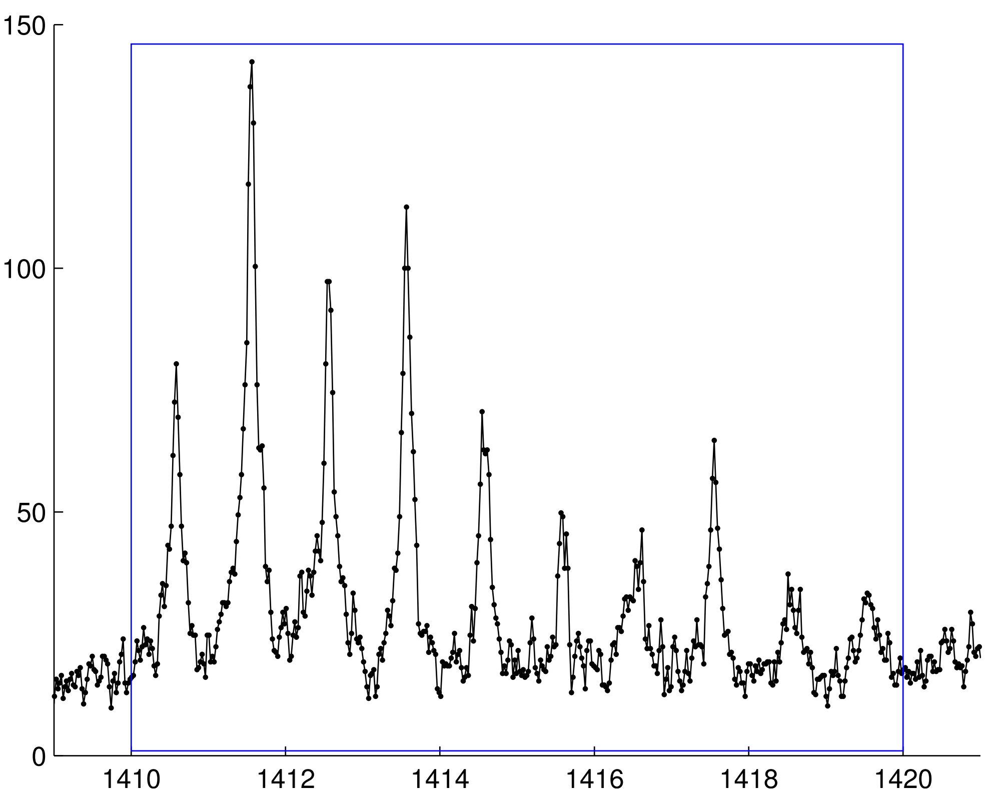

150
100
50
0
1410
1412
1414
1416
1418
1420
