# Supplementary material for: Isotope pattern deconvolution for peptide mass spectrometry by non-negative least squares/least absolute deviation template matching
Source: BMC Bioinformatics. 2012 Nov 8;13:291. doi: 10.1186/1471-2105-13-291 (PMC3608065; doi:10.1186/1471-2105-13-291)
Supplement: Additional file 3 — Evaluation and results. [file 1471-2105-13-291-S3.zip › eval/manual_annotation/excludedregions/screenshots/maldi/excludedreg_myo500_1.pdf]

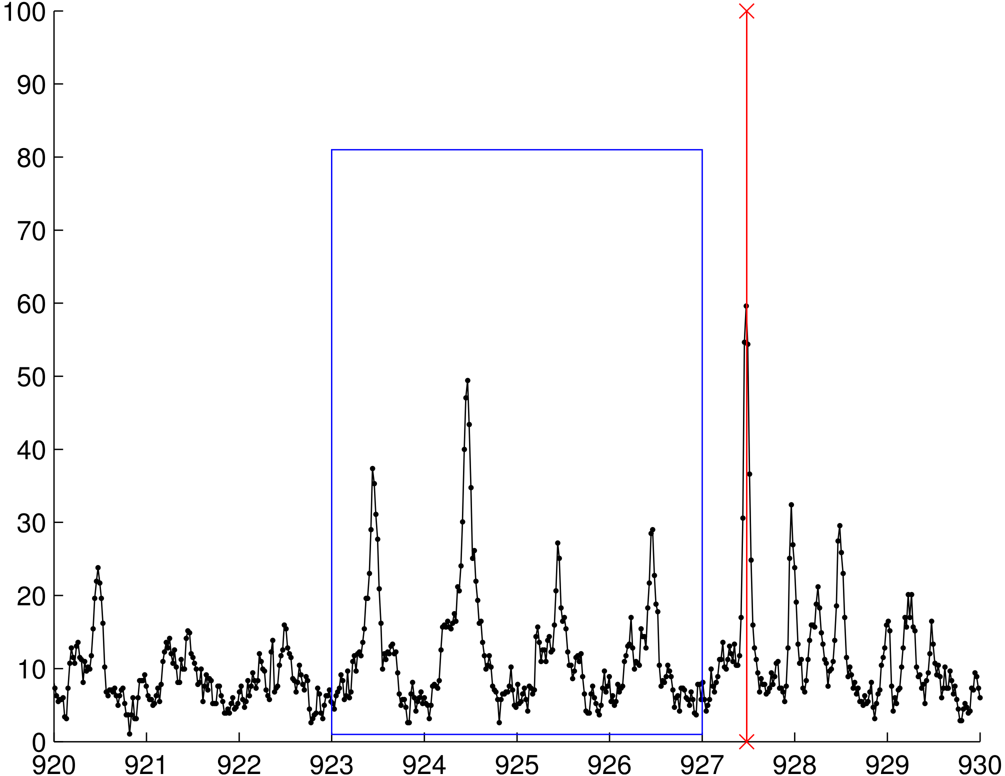
100
90
80
70
60
50
40
30
20
10
0
920
921
922
923
924
925
926
927
928
929
930
