# Supplementary material for: Isotope pattern deconvolution for peptide mass spectrometry by non-negative least squares/least absolute deviation template matching
Source: BMC Bioinformatics. 2012 Nov 8;13:291. doi: 10.1186/1471-2105-13-291 (PMC3608065; doi:10.1186/1471-2105-13-291)
Supplement: Additional file 3 — Evaluation and results. [file 1471-2105-13-291-S3.zip › eval/manual_annotation/excludedregions/screenshots/maldi/excludedreg_lys500_5.pdf]

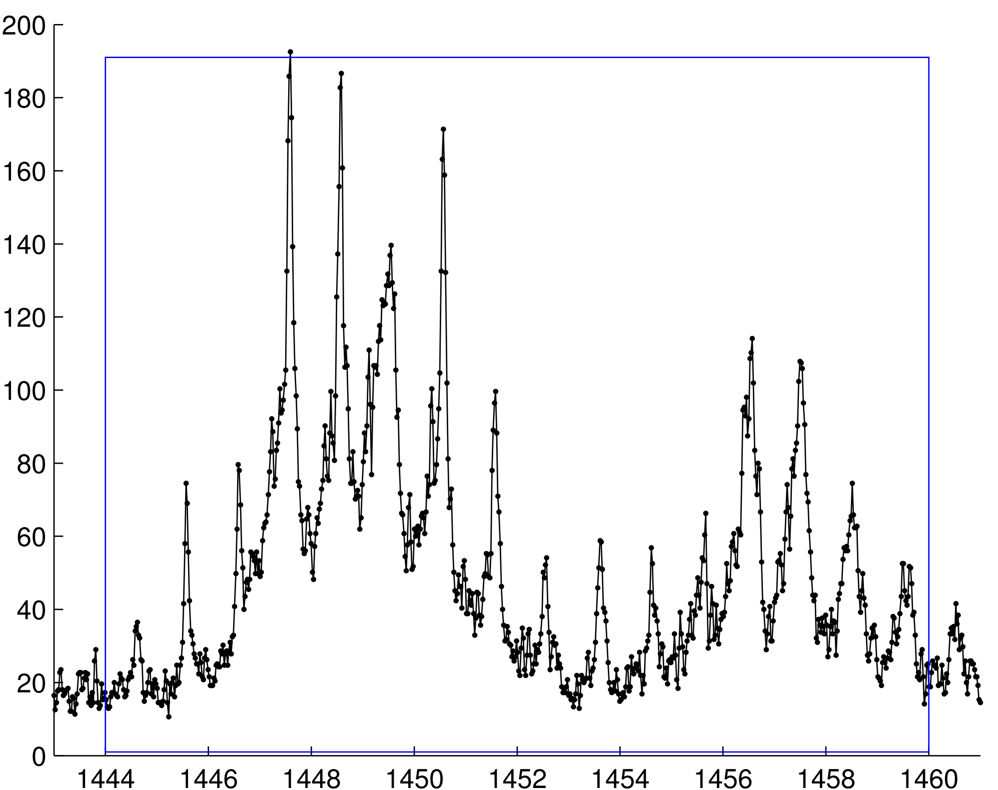
200
180
160
140
120
100
80
60
40
20
0
1444
1446
1448
1450
1452
1454
1456
1458
1460
