# Supplementary material for: Isotope pattern deconvolution for peptide mass spectrometry by non-negative least squares/least absolute deviation template matching
Source: BMC Bioinformatics. 2012 Nov 8;13:291. doi: 10.1186/1471-2105-13-291 (PMC3608065; doi:10.1186/1471-2105-13-291)
Supplement: Additional file 3 — Evaluation and results. [file 1471-2105-13-291-S3.zip › eval/roc/maldi/precrecplot_myo10_rev3_nofilter.pdf]

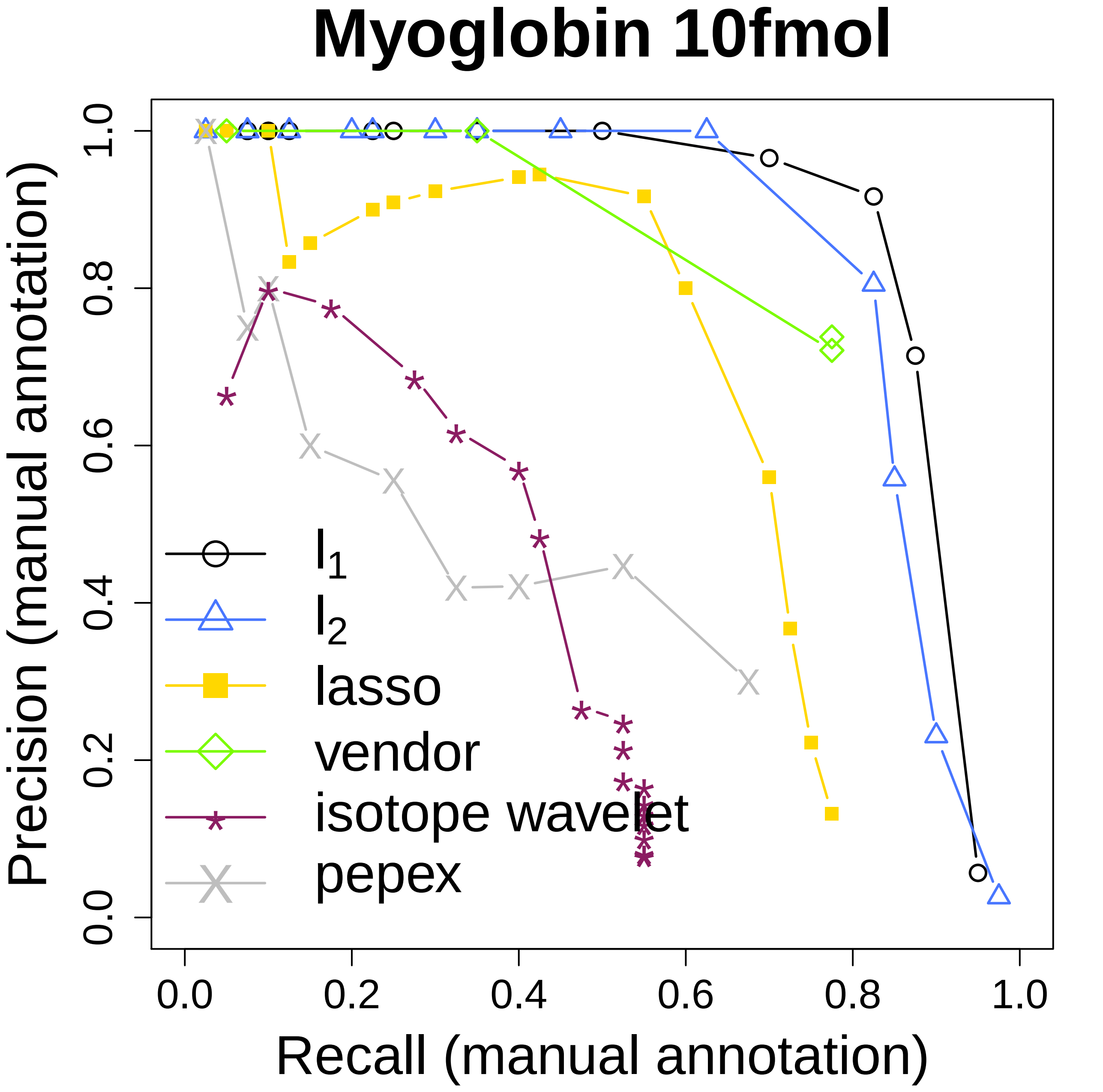

Myoglobin 10fmol
Precision (manual annotation)
Recall (manual annotation)
0.0
0.2
0.4
0.6
0.8
1.0
0.0
0.2
0.4
0.6
0.8
1.0
l1
l2
lasso
vendor
isotope wavelet
pepex
